# The neural underpinnings of vicarious experience

**DOI:** 10.3389/fnhum.2014.00384

**Published:** 2014-06-03

**Authors:** Bernadette M. Fitzgibbon, Jamie Ward, Peter G. Enticott

**Affiliations:** ^1^Monash Alfred Psychiatry Research Center, Central Clinical School, Monash UniversityMelbourne, VIC, Australia; ^2^Department of Psychology, University of SussexBrighton, UK; ^3^Cognitive Neuroscience Unit, School of Psychology, Deakin UniversityMelbourne, VIC, Australia

**Keywords:** social neuroscience, social cognition, mirror neurons, empathy, touch, pain, personality, vicarious experience

In recent decades there has been an explosion of empirical research into the social cognitive processes that underlie our social interactions. Coinciding with, or perhaps driving, the interest within this area is the development of modern neuroscientific techniques bringing real world experiences into the laboratory to produce biological models of how we experience and interact with other people.

In this research topic, we present a range of expert manuscripts that focus on one primary aspect of social cognition: the ability to recognize, understand and, in some cases, “feel” the experience of another person. To date, neuroscience research in this area has identified shared neural networks whereby we process another's action, emotion or sensation through overlapping brain regions as if we were carrying out that same action or experiencing that same emotion or sensation. Intriguingly, this research has shown that such vicarious activation of brain networks can span from an automatic and unconscious process through to an overt experience of the emotion or sensation of that observed in another person.

By investigating vicarious processes as well as exploring the influence of interpersonal characteristics such as empathy, we are taking a step toward better understanding the relationship between the social brain and social behavior. This includes the decision to make a pro-social response vs. fleeing potentially dangerous, or even socially awkward situations, such as witnessing another person embarrass themselves. Moreover, this research area has substantial implications for understanding disease and improving treatment options for people who experience psychiatric or neurological illness including autism spectrum disorder, where impairment in aspects of social functioning is a key feature. However, even beyond disorders where social function may be diagnostic, social impairments and difficulties in social relationships can have substantial functional consequences, as is often reported in illnesses such as depression and schizophrenia. Ultimately, understanding the neurobiology of social processes will provide the platform for targeted and appropriate treatment interventions.

In the work that follows, this research topic brings together a number of opinions, perspectives, hypotheses and theories, general commentaries, reviews and original research articles. Several key themes can be identified ranging from:

1. Definitional considerations including the distinction between vicarious and empathic experiences (Paulus et al., 2013), and why overt vicarious experiences may not represent a new form of synaesthesia, where sensory input in one domain results in a sensory experience in another (Rothen and Meier, 2013);
2. Exploration of vicarious shared neural experiences in the general population from physical touch and injury (Bufalari and Ionta, 2013) through to ostracism (Wesselmann et al., 2013) and how vicarious experience may differ between people according to attention (Morelli and Lieberman, 2013), interpersonal and personality differences (Schaefer et al., 2013; Vandenbroucke et al., 2013) such as empathy, and the influence of psychopathic (Marcoux et al., 2013) or autistic (Cooper et al., 2013) traits. Additional modulating factors of vicarious experience are also considered including expertise seen in physicians (Newton, 2013), the influence of the mother-child bond (Manini et al., 2013), the experimental administration of oxytocin and the effect of visual orientation (i.e., self vs. other) (Burgess et al., 2013).
3. The investigation of atypical vicarious experiences in the general population such as shared touch (Banissy and Ward, 2013) and pain and how feeling the pain of others may be linked with self-other confusion and prior pain experience (Derbyshire et al., 2013). Through to a physiological study exploring the experience of misophonia, describing a sensitivity to sound that can substantially limit ones ability to interact with others (Edelstein et al., 2013), and a commentary of why vicarious perception may drive contagious scratching (Ward et al., 2013);
4. The discussion of vicarious experiences in atypical populations including evidence against an impairment in shared neural networks in ASD (Enticott et al., 2013) and an argument for how models of vicarious pain experience may help us understand the relationship between core ASD symptoms better (Fitzgibbon et al., 2013);
5. Finally, the role of vicarious experience including vicarious motor system activation in understanding the behaviors of others (Avenanti et al., 2013) and how group membership may influence such processing and influence how we interact with others (Eres and Molenberghs, 2013).

Taken together, this research topic presents cutting edge research in a growing field which, while by no means definitive, represents exciting developments in the neurobiology underlying sharing experiences with others.

## Conflict of interest statement

The authors declare that the research was conducted in the absence of any commercial or financial relationships that could be construed as a potential conflict of interest.
